# Gibbons aren’t singing in the rain: presence and amount of rainfall influences ape calling behavior in Sabah, Malaysia

**DOI:** 10.1038/s41598-020-57976-x

**Published:** 2020-01-28

**Authors:** Dena J. Clink, Abdul Hamid Ahmad, Holger Klinck

**Affiliations:** 1 Center for Conservation Bioacoustics, Cornell Laboratory of Ornithology, Cornell University, 159 Sapsucker Woods Road, Ithaca, NY 14850 USA; 2 Institute for Tropical Biology and Conservation, Universiti Malaysia Sabah (UMS), Kota Kinabalu, Sabah Malaysia

**Keywords:** Animal behaviour, Behavioural ecology

## Abstract

Early morning calling occurs across diverse taxa, which may be related to optimal conditions for sound transmission. There exists substantial inter- and intra-specific variation in calling time which is influenced by intrinsic, social and/or environmental factors. Here, we investigate environmental predictors of calling in gibbons. We hypothesized that male solos— which occur earlier and tend to be longer than duets—would be more influenced by environmental variables, if earlier, longer calling bouts are energetically costly, and therefore limited by overnight energy expenditure. Our top model for male solo events included amount of rain in the previous 24 hours, and explained 30% of the variance, whereas the top model for duet events (which included presence and amount of rainfall) explained only 5% of the variance. Rain the previous night led to a later start time of male solos (~30 minutes), but our top model for duet start time did not include any reliable predictors. Male solo events appear to be more influenced by environmental factors, and duets may be influenced more by social factors. Our results are in line with previous studies that show that changes in overnight conditions —which may alter energy expenditure —can influence early morning calling behavior.

## Introduction

Animals in diverse habitats ranging from tropical forests to deserts engage in early morning calling activity, and understanding the drivers for synchronized calling in the early morning hours has garnered much interest among ornithologists and primatologists. The function of most early morning vocalizations is thought to be either for territorial communication or mate attraction^1^. It has been proposed that animals call in the early morning because the conditions for sound transmission are best^2^, but within the early morning calling activity there is a substantial amount of inter- and intra-specific variation in call timing. Understanding the selective pressures that lead to variation in timing of animal calling remains an open question^3^, but variation in call timing is most likely the result of a combination of species - and individual - specific intrinsic, social and environmental factors^4^.

The main abiotic factors shown to influence sound attenuation across habitats are the topography, temperature profile, wind direction and speed, humidity, and rainfall^5^. In addition, other factors that may not be directly related to sound transmission can also influence timing of the dawn chorus, such as moonlight at dawn or the time of sunrise. In a temperate bird community in Ontario, Canada the timing of the dawn chorus was correlated with amount of moonlight at dawn, cloud cover and precipitation^6^. Near the equator, changes in photoperiod are minimal, but dawn singing in male silver-beaked tanagers (*Ramphocelus carbo*) in the equatorial lowland Amazonas was shown to be correlated with even slight increases in day length^7^. Precipitation may influence calling behavior due to the masking effects of the sound of rain, or it could be linked to an overall decrease in animal activity during rainy periods^8^.

In addition to environmental selection pressures, variation in calling behavior may also be related to energetic costs. Across diverse taxa, calling rates can be linked to individual metabolic rate, such that call features (including calling rate) vary predictably with body size and temperature^9,10^, providing evidence that calling can be metabolically costly. In birds, the energetic costs of singing were shown to be relatively low compared to other daily activities^11^, but birds with more complex songs were shown to have higher energy requirements than birds with less complex songs^12^. There is also evidence that animals will modify or reduce their calling activity in response to environmental changes. For example, European robins (*Erithacus rubecula*) were experimentally subjected to warm or cold overnight temperatures, and those subjected to cold temperatures were less likely to initiate vocal behaviors^13^. In silvereyes (*Zosterops lateralis*), males sang the dawn chorus for a greater proportion of the time after receiving supplementary food, and supplementary food also led to an increase in song complexity^14^. Male simakobus (*Simias concolor*), an old world monkey endemic to the Mentawi islands off Sumatra, avoided calling during the hottest times of day, and also emitted shorter calls during hot periods, providing evidence that calls in nonhuman primates may also be influenced by thermoregulation costs^15^.

Acoustic communication is ubiquitous in non-human primates, and acoustic signals often mediate spatial proximity, particularly in territorial primates^16^. Duetting occurs only in one ape family (Hylobatidae; hereafter referred to as gibbons^17^). In addition to engaging in coordinated duets, male gibbons also engage in solo songs irrespective of their paired or floating status^16,18^. Although the function of duets across taxa remains a topic of debate^19^, male solos and duets in gibbons appear - at least in part - to convey information to conspecifics about the caller(s), whether it is for mate attraction in the case of unmated males^20^, to advertise the pair-bond status^21,22^, for territorial defense^16,23^, to advertise individual and/or pair resource holding potential^24^, or provide information regarding individual identity^25–28^ or condition^24,29^. Although the energetic costs of calling in gibbons have not been quantified, available evidence suggests that performance of calling bouts may be related to energy balance and expenditure, particularly in males^30^. A meta-analysis of 21 gibbon populations (across nine different species) found that when fruit availability was reduced, frequency or duration of calling bouts was also reduced, and this effect was greater in males, and particularly males at higher latitudes, where costs of thermoregulation are assumed to be higher^30^.

Gibbons tend to call in the early morning hours^18^, but generally do not engage in a dawn chorus as many songbirds do, where all individuals in a particular area call at the same time, although there is a cascading effect wherein if one pair starts calling others in the population often start to call^31^. It has been noted that gibbons call less after rainy nights, with most acoustic survey methods recommending that gibbon surveys be conducted only after dry, rainless nights^32^, and rainfall has been identified as one of the main factors negatively influencing gibbon calling across species^16,31–33^, except in black-crested gibbons (*Nomascus concolor jingdongensis*)^34^. This observed reduction in calling may be related to energetic costs of thermoregulation^9,10^, reduced suitability for sound transmission^35^ or a behavioral response to cold and wet conditions^8^. It is also possible that increased rainfall is correlated with lower ambient temperatures, which leads to increased energy requirements, requiring that gibbons spend more time foraging and less time calling^31^.

In Kloss gibbons (*Hylobates klossii*), rainfall during the period in which animals normally call inhibited calling by both males and females–which the authors attribute to a reduction in acoustic communication space on rainy days–but overnight rainfall and low temperatures did not influence calling^33^. Male Kloss gibbons were actually more likely to call on mornings with lower temperatures. Agile gibbons (*H. albibarbis*) in Central Kalimantan, Indonesia had a lower probability of calling on days with rain, and if they did call they started calling later^31^. Reports on the effects of temperature on gibbon calling behavior have been inconsistent. For example, Eastern hoolock gibbons (*Hoolock leuconedys*) had a higher probability of calling on days when mean morning temperature was higher, and these gibbons live in the coldest reported gibbon habitats (annual mean temperature of 13.0 °C) where energy costs associated with thermoregulation are assumed to be high^34^. In contrast, colder temperatures did not inhibit singing in white-cheeked gibbons (*Nomascus* spp.), with white-cheeked gibbons calling more during the cold, dry season^36^. In agile gibbons, there was no effect of temperature on calling behavior^31^. Documented differences in response to environmental variables may be related to phylogeny, variation in temperature extremes across sites, or even site-specific factors, as studies of the same species have reported different results^36,37^. Previous studies of the influence of environmental factors on gibbon calling behavior focused on a small number of habituated groups^16,31,34,37–39^ (but see^33^), and the results may not be representative of population-level patterns of behavior.

Here, we aim to investigate the proximate causes of calling behavior in a population of Bornean gibbons (*H. muelleri*) in Danum Valley Conservation Area, Sabah, Malaysia using passive acoustic monitoring (PAM) which utilizes battery-operated autonomous recorders to continuously monitor the soundscape in the deployment area. Bornean gibbons are pair-living, territorial hominoids which regularly engage in species-specific coordinated duets between the male and female of the pair, with the female contribution to the duet being longer and more elaborate than the male contribution^17^. We hypothesized that gibbon calling behavior would be non-random with respect to environmental predictor variables including rainfall, temperature and lunar phase^31^. We also predicted that male solos would vary more predictably with environmental variables than duets, given previous reports documenting a strong influence of the availability of high-energy foods and latitude (a proxy for energy costs of thermoregulation) on male calling bouts^30^. In addition, in this species male solos tend to start earlier in the morning and last longer than duets^16,18^, which are both factors that may lead to solos being more energy-limited than duets^30^.

Specifically, we predicted that: 1) the probability of gibbon calling events would decrease if there was rainfall the night before and if overnight temperatures were lower; 2) lunar phase would influence the start time of gibbon calls, and the presence of moonlight in the earlier morning hours would facilitate earlier calling; and 3) the environmental effects on calling behavior would be stronger for male solos than for duets. A clear understanding of patterns of calling behavior, and the influence of environmental predictors on calling, is important for effective planning and interpretation of acoustic surveys^36^, and we highlight the potential for PAM to answer questions about the behavioral ecology of vocal primates at larger spatial and temporal scales than is possible with the use of human observers.

## Results

### Gibbon calling behavior in Danum Valley Conservation Area

We report the results of the analysis of 14,784 hours of continuous recordings across 11 recording locations and 250 gibbon calling events (73 male solos and 177 duets; Table 1). Our method did not allow us to distinguish between calling individuals, so the results we report are at the population-level. All of the calling events occurred between the hours of 05:00–11:00 local time (LT). The mean duration for male solos was 41.4 minutes (range = 6.5–88.1 min) and the mean duration for duets was 15.1 minutes (range = 1.6–55.4 min). Male solos tended to occur earlier in the morning (median start time of 05:46) and duets occurred later (median start time of 07:38; Fig. 1). The earliest solo occurred at 05:00, and the earliest duet occurred at 06:00.

**Table 1:** Total number of recording days, hours and number of calling events summarized by recorder. Recorder S10 was deployed later in the field season, which is why it has fewer recording days, and the variable recording days and hours for the remaining recorders are due to differences in battery-life and/or unit functioning.

Recorder	Recording Days	Recording Hours	Number of Male Solos	Number of Duets	Total Events
S10	21	504	4	9	13
S11	60	1,440	8	25	33
S12	67	1,608	3	14	17
S13	49	1,176	0	9	9
S14	68	1,632	5	34	39
S15	65	1,560	10	6	16
S16	54	1,296	2	13	15
S17	43	1,032	8	10	18
S18	48	1,152	6	6	12
S19	96	2,304	21	26	47
S20	45	1,080	6	25	31
TOTALS	616	14,784	73	177	250

**Figure 1:**
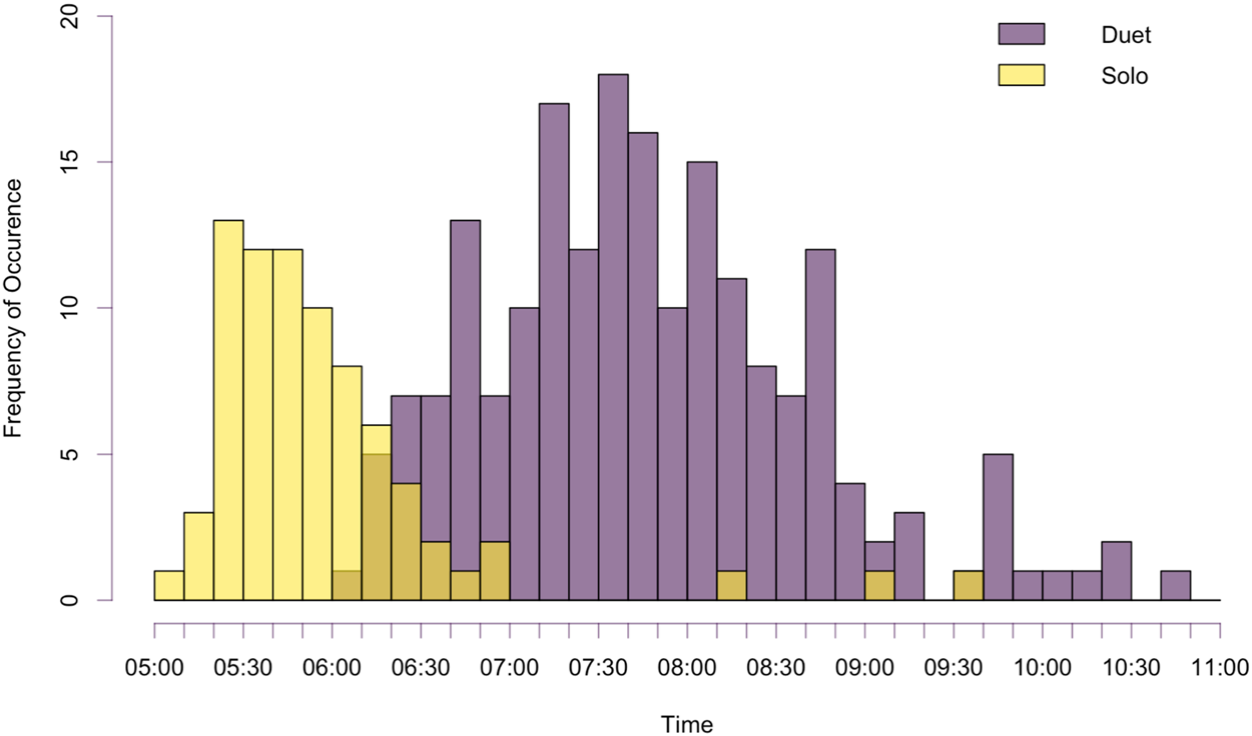
Start times of Bornean gibbon male solos and duets in Danum Valley Conservation Area, Sabah, Malaysia from February to July 2018. The color not shown in the legend indicates periods of overlap in which both solos and duets occurred during that time period.

### Environmental predictors of gibbon calling events

**Figure 2:**
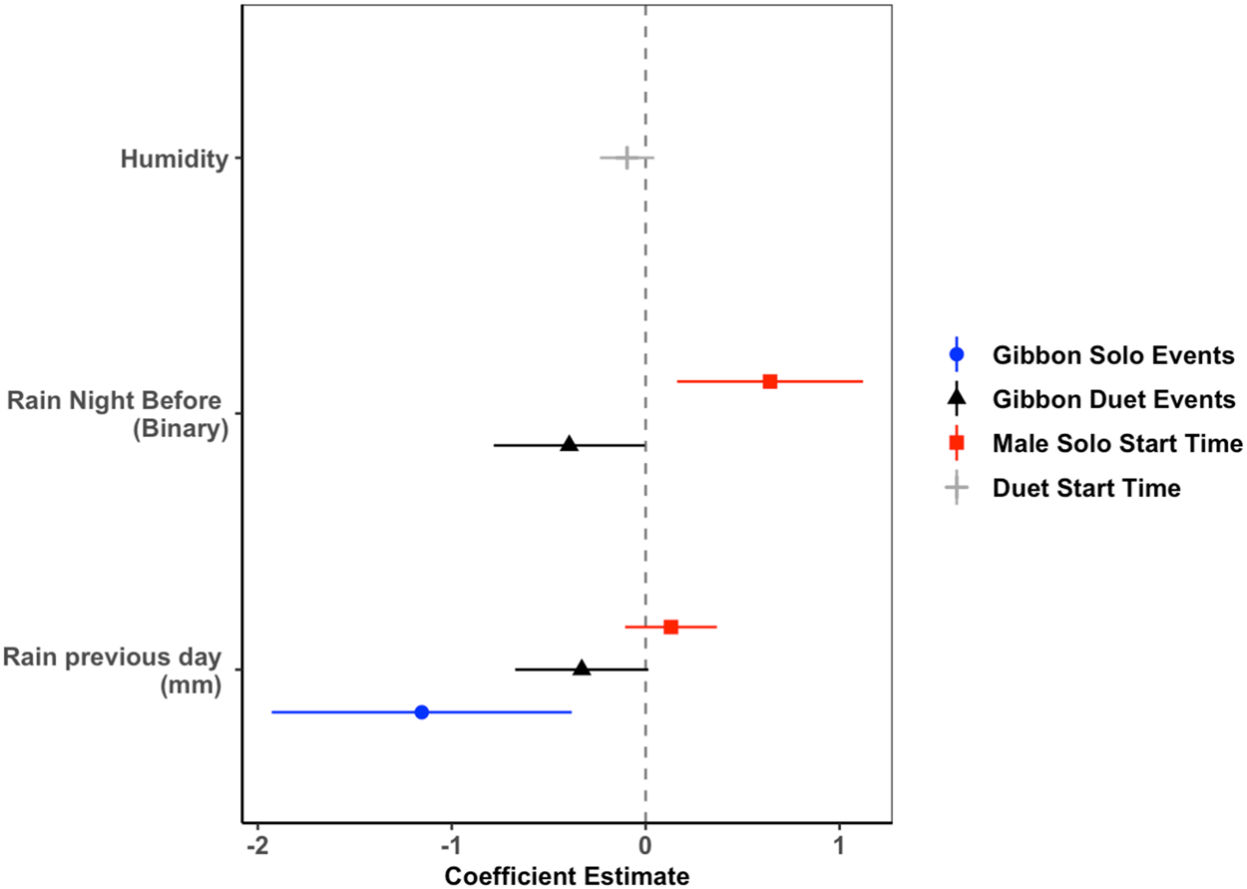
Coefficient estimates (along with 95% confidence intervals) for top models predicting gibbon calling events (either male solos or coordinated duets) and start time of solos or duets. For solo events, the amount of rain the preceding night had a reliably negative effect, and for duets presence of rain the preceding night and the amount of rain both had a negative effect. For male solo start time, presence of rain the preceding night had a reliably positive effect. The top model for duet start time included humidity, but we did not consider this a reliable predictor as the 95% confidence interval overlapped zero. Both solo and duet start times were scaled for better visualization. Coefficient plots such as these represent the direction and reliability of the estimate, but not the effect size. We considered predictors reliable if the 95% confidence intervals did not overlap zero^40^.

Using a model selection approach wherein each model represents a specific hypothesis, and model comparison using Akaike information criterion (AIC), we found that our top model for presence of male solo events comprised 45% of the model weight and included one predictor variable: the amount of rain (mm per 24 hours) the previous day which had a reliably negative effect on male solo events (estimate = −1.15, se = 0.40). The second highest ranked model for presence of male solo events comprised 38% of the model weight and included a binary predictor for the presence of rain between 18:00–06:00 the previous night (estimate = −0.38, se = 0.29), and amount of rain the previous day (estimate = −0.95, se = 0.41). Combined, the two top models for male solo events comprised 83% of the model weight and performed substantially better than the intercept only model (ΔAICc = 12.3, 0% model weight; Table 2). We calculated a pseudo-R^2^ value and found that the predictor in the top model explained 27% of the variance in male solo events, and the entire model (predictor variables plus random effects) explained 32% of the variance. Coefficient estimates for the top model for male solo events are shown in Fig. 2.

**Table 2:** Akaike’s information criterion (AICc) model comparison results showing the top two models and the intercept-only model for gibbon calling events, male solo start time and duet start time. All models included a random intercept for recorder.

	Model	logLik	AICc	dAICc	df	weight
Gibbon calling events (solos) ~	rainfall previous day (mm/24 h)	−200.07	406.18	0.00	3.00	0.44
	night rain + rainfall previous day (mm/24 h)	−199.22	406.51	0.33	4.00	0.38
	intercept only	−199.19	408.48	2.30	5.00	0.14
Gibbon calling events (duets) ~	night rain + rainfall previous day (mm/24 h)	−358.31	724.68	0.00	4.00	0.47
	night rain + rainfall previous day (mm/24 h) + minimum temperature	−360.28	726.59	1.91	3.00	0.18
	intercept only	−358.30	726.69	2.00	5.00	0.17
Male solo start time ~	night rain + rainfall previous day (mm/24 h)	−347.38	705.75	0.00	5.00	0.30
	night rain + minimum temperature	−347.52	706.03	0.28	5.00	0.26
	intercept only	−346.68	706.77	1.02	6.00	0.18
Duet start time ~	humidity (%)	−272.06	552.34	0.00	4.00	0.22
	rainfall previous day (mm/24 h)	−272.57	553.34	1.01	4.00	0.13
	intercept only	−271.69	553.71	1.37	5.00	0.11

**Figure 3:**
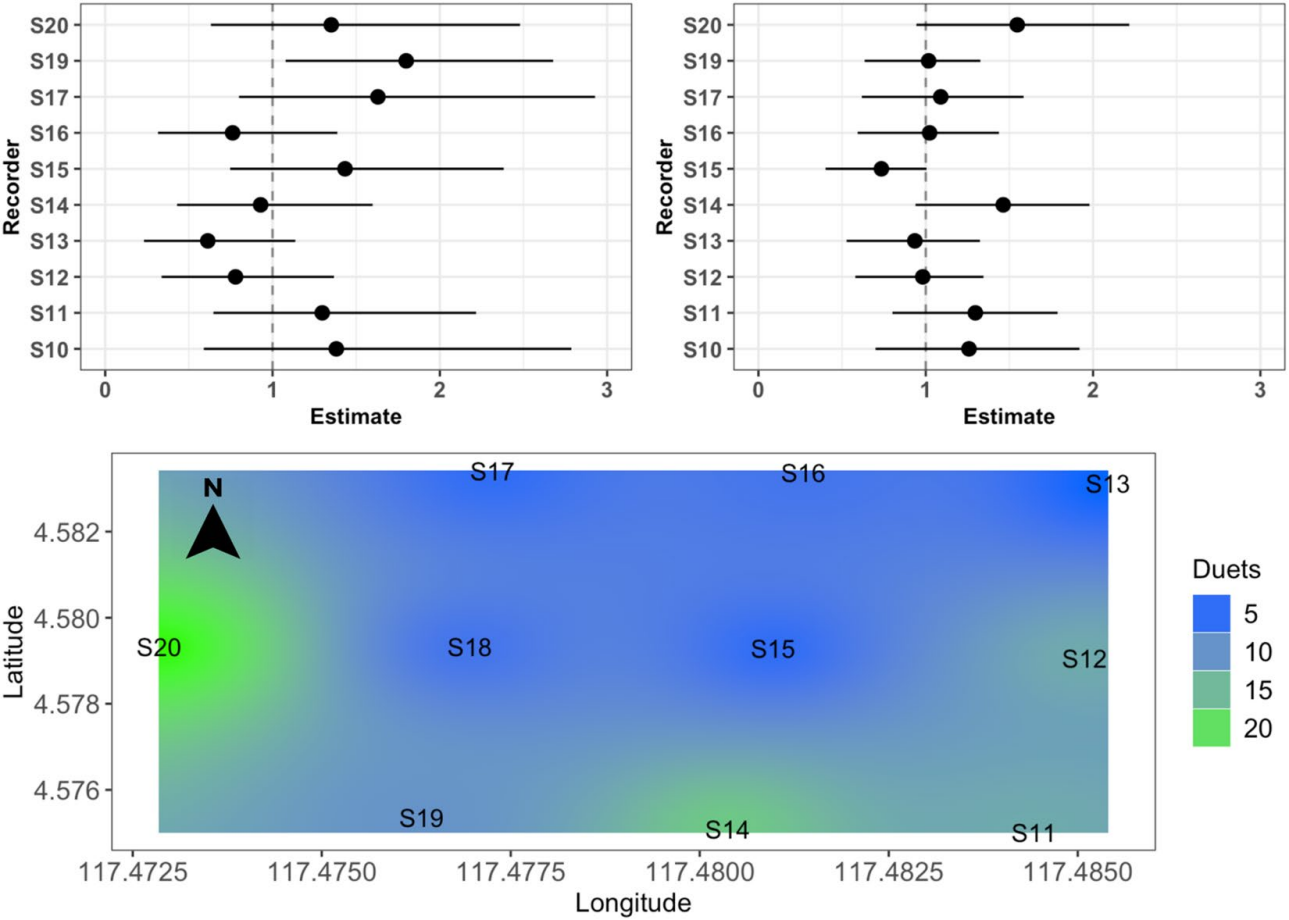
Conditional modes for gibbon solos (top left) and duets (top right) at each recording location within Danum Valley Conservation Area, Sabah, Malaysia, and gibbon duet event density across the 3 km^2^ study area for February-March 2018 (bottom). Top: Conditional modes (dots) that are further from zero are less like the average response, and wider confidence intervals indicate greater variance for that particular recording location. We considered recording locations where the 95% confidence intervals do not overlap zero^40^ to have gibbon calling events that differ significantly from the average. Bottom: Duet event density (n = 103 calling events) was calculated for the time period of February-March 2018 when all recorders were recording simultaneously using inverse distance weighed interpolation^41^ with the R package ‘gstat’^42^. Recorders were deployed on a grid at approximately 750-m spacing. Recorder S10 was deployed later in the field season, so therefore was not included in the calling event density map.

Our top model for duet events included a binary predictor for the presence of rain the night before (estimate = −0.40, se = 0.20), and amount of rain the previous day (estimate = −0.33, se = 0.17) and comprised 47% of the model weight. The top model performed substantially better than the intercept only model (ΔAICc = 9.3, 0% model weight; Table 2), but the pseudo-R^2^ values indicate that the predictors in the top model explained less than 5% of the variance, and the entire model (predictors plus random effects) explained only 8% of the variance. Coefficient estimates for the top model for duet events are shown in Fig. 2. There were substantial differences between recorders in both solo and duet events (Fig. 3), with some recording locations having more gibbon calling events than expected, and others having fewer, indicating that there is spatial variation in gibbon calling across our site. See discussion for possible explanations regarding spatial variation in calling events at our site.

### Environmental predictors of gibbon calling time relative to nautical dawn

**Figure 4:**
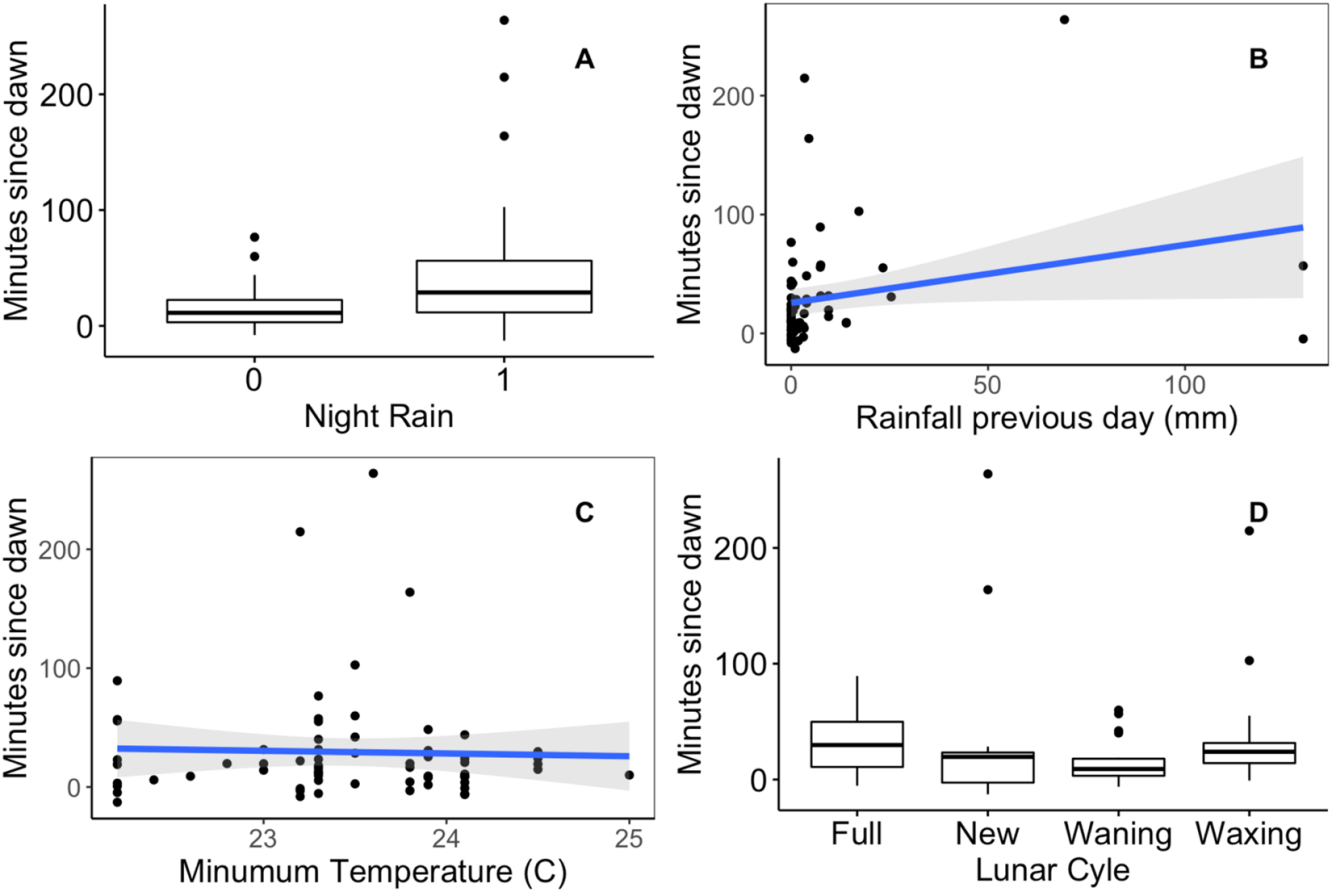
Relationship between male solo start time (relative to nautical dawn) and rain the night before (**A**), rainfall in the previous 24 hours (mm per 24 hours) (**B**), minimum temperature (**C**) and lunar cycle (**D**). The top model for male solo start time included the binary variable for presence of rain the night before, but did not include amount of rainfall, minimum temperature or lunar cycle. See text for discussion of extreme values.

For male solo start time, we found that the binary variable indicating rainfall the night before led to a later start time by approximately 30 mins (estimate = 30.26, se = 11.53; Fig. 2 and Fig. 4A), and amount of rain the previous day also had a reliably positive effect on male solo start time (estimate = 6.17, se = 5.70; Fig. 4B), but minimum temperature (Fig. 4C), moon cycle (Fig. 4D) and humidity did not influence male solo start time. The top model which included night rain and amount of rain the previous night comprised 30% of the model weight, and the second top model which contained night rain and minimum temperature (which was not a reliable predictor; estimate = 5.40, se = 5.72) comprised 26% of the model weight, with both models (combined 56% of model weight) performing substantially better than the intercept only model (ΔAICc = 8.4, 0.01% model weight; Table 2). The predictor variables explained 15% of the variance in male solo start time, with all of the variance explained by the predictors. Coefficient estimates for the top model for male solo start time are shown in Fig. 2. For duet start time, the top model comprised 24% of the model weight and included humidity (estimate = −0.10, se = 0.07; Fig. 2), but this model did not perform substantially better than the intercept only model (ΔAICc = 1.7, 0.01% model weight; Table 2), and the pseudo-R^2^ values indicate that the predictor explained less than 1% of the variance, and the entire model explained only 3% of the variance.

### Reanalysis with subsets of data

There were three instances of male solos that were much later than the others (>100 mins after dawn) (>100 mins after dawn; Fig. 1), and we were interested to see if those late solo start times had a disproportionate influence on the inference from our models, so we re-ran the model selection removing those three data points. We found that removing the extreme values did not have a substantial influence on model inference for male solo event occurrence, as the top model comprised 40% of the model weight, performed substantially better than the intercept only model (ΔAICc = 12.3, 0.00% model weight) and included two predictors, the presence of rain the night before (estimate = −0.42, se = 0.29), and amount of rain the previous day (estimate = −0.05, se = 0.02). For male solo timing, we found that the top model included only one predictor variable, the binary variable indicating rainfall the night before (estimate = 19.63, se = 6.99), and the top model accounted for 61% of the model weight, which was slightly better than the intercept only model (ΔAICc = 5.2, 0.05% model weight).

There were two occasions when rainfall the previous night exceeded 100 mm (Fig. 4B), and we were interested to see if those two points had a substantial influence on model inference for male solo timing. We found that the top model for male solo timing (which accounted for 53% of model weight) included the binary variable indicating presence of rain the previous night (estimate = 28.11, se = 8.88), meaning that on average if there was rain the previous night, males started their solos 28 minutes later minutes later. The pseudo-R^2^ values for male solo timing (with extreme values removed) indicate that the predictor in the top model explained 13% of the variance, and the entire model (predictors plus random effects) explained 16% of the variance.

## Discussion

The main goal of this study was to investigate the environmental predictors of gibbon calling behavior in Danum Valley Conservation Area, Sabah, Malaysia. We found that amount of rainfall in the previous 24 hours was a reliably negative predictor of male solo events, and that presence of rain the previous night led to an approximately 30 minute later start time of male solos. We did not find that minimum overnight temperature or lunar cycle had a reliable effect on gibbon calling events or male solo start time. For duet events, although both presence of rain the previous night and amount of rainfall in the previous 24 hours were included in the top model, the model explained less than 10% of the variance in duet events, indicating that inferences from this model are not ‘useful or reliable’^43^ and for duet timing, the model containing humidity as a predictor variable was ranked highest, but this model explained less than 1% of the variance in duet timing. Importantly, our results are in line with previous reports of calling behavior of other species in the genus *Hylobates* but may not be applicable to other genera. For example, contrary to reports on *Nomascus* spp. wherein most duets occur within 20 minutes of sunrise^36^, we found a high degree of variability in duet start time with the majority of duets occurring between 06:00–09:00, which is in agreement with previous reports for this species^16^. We show that PAM has the potential to provide important insights into behavior of vocal primates, without the need for habituation, which can be time-consuming and potentially alter the vocal behavior of focal animals^34^.

Our results partially supported our predictions, particularly in reference to male solos, as we predicted that since solos start earlier and tend to be longer in this species, that solos would be more constrained by energetic and/or thermoregulatory costs. The selection pressures that led to differences in start time between male solos and duets are unclear, but since male solos tend to start earlier, they are more likely to be influenced by overnight weather conditions than duets. Previous reports of soloing by paired versus floating males indicate that performances by solitary males are ‘indistinguishable’ from those of paired males^18^, which means that we have no way to know whether the solos we documented in our dataset paired or floating males mated or solitary males. Although the function of gibbon solos and duets remains a topic of debate^44^, if the male solo functions to advertise resource holding potential^24^, as an assessment signal^30^ or for mate attraction^45^, it is possible that mated or unmated males are differentially impacted by environmental conditions. Whereas if solos simply serve a function to provide information to conspecifics regarding individual identify and location^44,46^, we would not expect to see differences between paired versus floating males. Our results are in line with studies on other taxa that indicate overnight conditions which lead to an increase or decrease in energy expenditure over baseline can play an important role in influencing morning calling behavior^13,14^. We also found that there were substantial differences in gibbon calling events across recording locations at our site, which are likely due to variation in calling behavior across pairs and individuals where the recorders were placed, or spatial variation in occurrence or territory boundaries, and less likely related to habitat differences, as all of our recorders were placed in lowland, relatively pristine forest.

Rain can have a large influence on how sound travels through the environment. For example, tawny owl (*Strix aluco*) calls have a 69-fold advantage in communication space under dry versus rainy conditions, and tawny owls decrease calling during periods of rain^35^. But animal vocal animal response appears to be taxon specific, as túngara frogs (*Physalaemus pustulosus*) enhanced calling rate and complexity under rain noise playback treatments when compared to control and wind noise treatments^47^. The presence of rain can also alter the behavior of animals. Rain noise delayed the time of bat emergence from their roosts^48^, and two primate species (howler monkeys, *Alouatta seniculus*, and spider monkeys, *Ateles belzebuth*) visited mineral licks less often on days that were cloudy or rainy^8^. Here, we show that the presence of rain on the preceding night led to fewer overall gibbon calling events, and also led to a later start time for male solos. Given the difficulties with isolating calls during rain events from the LTSAs we did not include any calling bouts during actual rain events. Although gibbons tend to call less during actual periods of rain (Clink, personal observation), with PAM it is difficult to know whether there is a decrease in gibbon calling events because they are calling less, or because gibbon calls are harder to detect in noisy recordings when there is a lot of rain. The presence of rain the preceding night is unlikely to have a large impact on how gibbon calls are transmitted through the forest, unless there is a significant drop in ambient temperature, so it seems that the decrease in calling events and later start time are better explained by behavioral modifications (i.e. energy conservation) as opposed to sound transmission.

Contrary to previous work documenting patterns of activity in cathemeral populations of owl monkeys^49^, and on bird dawn chorus start time^6,50^ we did not find that lunar cycle had an effect on gibbon start time. A meta-analysis of 57 species from 27 families of neotropical songbirds showed that foraging height (canopy versus ground) had a strong influence on timing of dawn chorus, and that ambient light was a strong predictor of singing, with canopy species singing earlier^51^. As male solos tend to start earlier in the morning, and in some cases before dawn, we predicted that male solo start time would be more influenced by lunar cycle than duets. A limitation of our analysis is that we used moon phase (new, waxing, full and waning) as a categorical predictor variable, and were not able to use measures of ambient luminance. It is possible that moon phase was not correlated with ambient luminance, and that presence of clouds masking available moonlight confounded our results. Further studies which incorporate measures of ambient luminance will be informative.

Although it is evident that gibbon calling behavior is at least in part influenced by environmental factors, there is still substantial individual- or pair-level variation in calling behaviors. For example, at the population-level, gibbon pairs tend to call less after a rainy night, however, some pairs will still call. We showed that there were differences in gibbon calling events across our site, which may be related to spatial heterogeneity in habitat quality and gibbon density, or it may also reflect behavioral differences in individuals or calling pairs. Animals are known to exhibit distinct personality traits, or behavioral syndromes, where certain individuals are more bold or aggressive than others^52,53^, and it seems likely that certain gibbon individuals or pairs call more often than others, which would also lead to spatial variation in gibbon calling events. Previous reports of home range size of two gibbon pairs in our population were about 0.34 km^2,^ ^54^, but home range size can vary widely even within the same population^55^, making it difficult to know precisely how many pairs were included in the present study. Based on conservative estimates of gibbon home range size (0.3–0.5 km^2^) it seems likely that at least 6–10 pairs were recorded in our 3 km^2^ study area. Future studies which incorporate known pair-identity will improve understanding of the drivers of spatial variation in gibbon calling behavior.

Although we were able to get hourly rain data from the LTSAs, a limitation of the present study is that we were not able to obtain hourly temperature, humidity or cloud cover data, and we were also not able to get weather data for each specific recording location. Although it seems unlikely that there was much spatial heterogeneity in terms of weather across our relatively small study area, more precise measures of hourly temperature, humidity, cloud cover and ambient luminance would be informative. Nonetheless, we show that broad-scale weather variables and inspection of long-term spectrograms can provide important information, and data such as these may be useful for implementing acoustic monitoring programs across large temporal and spatial scales. Although not a limitation for the present study, as we were interested in population-level patterns of calling behavior, the identification of calling individual or pair using PAM remains a challenge. It is possible that our results were influenced by the calling behavior of a few individuals or pairs, which are not representative of the population, as we were not able to identify calling individuals, and future studies which incorporate the known identify of calling individuals or pairs will be informative.

For vocal primates such as gibbons, morning vocalizations are a fundamental component of their behavior, and quantifying variation in calling behavior has important implications for understanding their ecology and for conservation. Although common in studies of behavior in marine mammals^56^, the use of PAM in primates has been limited, and the majority of studies relying on autonomous acoustic monitoring of primates have focused mostly on occurrence or presence/absence of calling animals^57,58^, and less on the potential for PAM to improve our understanding of primate behavior (but see^59^). With the improvements in battery-life and data storage capabilities, the use of autonomous recorders has the potential to revolutionize how we study the social behavior of vocal animals^60^. The use of PAM to monitor gibbons - particularly in protected and inaccessible areas such Danum Valley Conservation Area - can provide important long-term baseline data on how calling behavior varies over space and time, and in relation to environmental variables, which can then be used to inform gibbon PAM programs in disturbed habitats.

Understanding the proximate and ultimate causes of variation in behavior are fundamental goals of evolutionary biology, and vocal communication systems provide a useful model to address these goals. Here we show how PAM can be used to investigate proximate factors that influence calling behavior in a population of Bornean gibbons, and we found that presence and amount of rainfall were both strong predictors of gibbon calling events and timing of male solos. Male solos appear to serve a different function from the coordinated duets, and the results of our study combined with previous studies^30,31^ provide evidence that gibbon calling events are influenced by overnight weather conditions, which may be related to overnight changes in energy expenditure. It appears that even within the Malaysian state of Sabah, there are population-level differences in call timing, with certain populations calling earlier than others (Clink, personal observation), which may be related to differences in food availability or population density, and further research comparing differences in calling behavior across populations will be necessary to understand these differences.

## Methods

### Study subjects

Mueller’s Bornean gibbons (*Hylobates muelleri*) are pair-living primates which engage in species- and sex-specific vocalizations, generally during the early morning hours. Male gibbons will engage in solo bouts whether they are floaters or in a mated pair, but only mated pairs engage in duets, wherein males and females emit alternating, coordinated and stereotyped vocalizations^18^. Each duet bout consists of multiple repeats of the distinct male and female contributions, and a previous study on Bornean gibbons in Kutai Game Reserve, East Kalimantan, Indonesia showed that duet bouts lasted for an average of 15 minutes, but there was substantial day to day variation in calling, and if a pair called on a particular day, they would often engage in more than a single bout, but often there were days where a pair would not call at all^16^.

### Acoustic data collection

**Figure 5:**
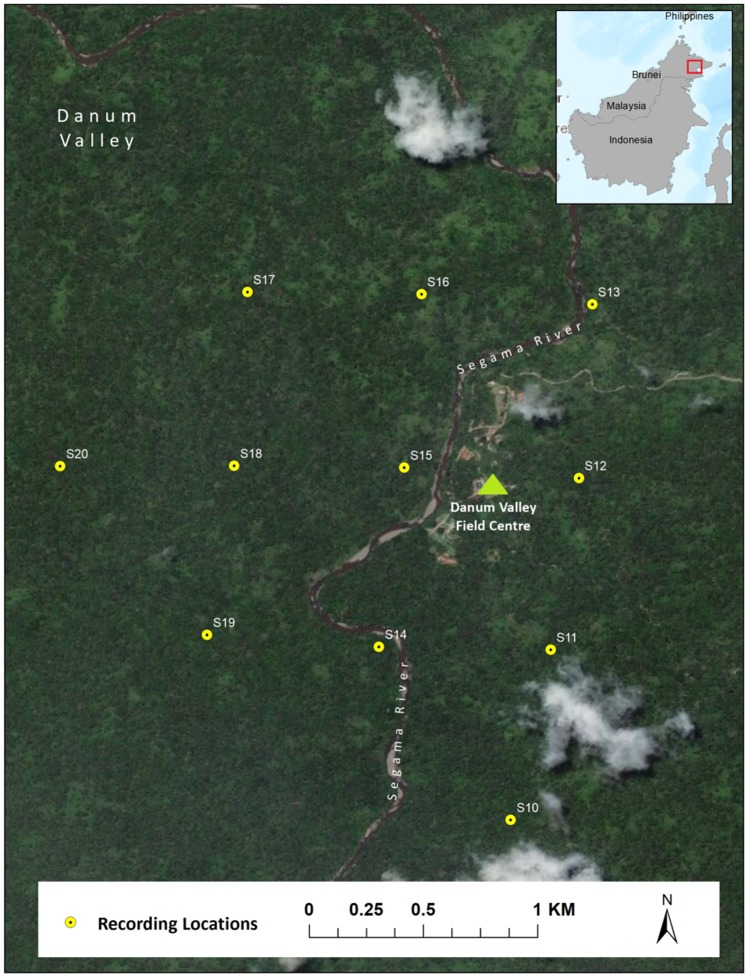
Map of autonomous recorder locations in Danum Valley Conservation Area, Sabah, Malaysia. Each point represents the location of a single Swift recording unit. Map was made using ArcGIS (ESRI) version 10.5.1 (www.esri.com).

We deployed eleven Swift autonomous recording units (https://www.birds.cornell.edu/ccb/swift/)^61^ spaced on a 750-m grid encompassing an area of approximately 3 km^2^ in the Danum Valley Conservation Area, Sabah, Malaysia (4°57′53.00′′N, 117°48′18.38′′E) from February – July 2018 (Fig. 5). The climate of Danum Valley has been defined as ‘aseasonal’^62^, lacking distinct wet and dry seasons, so there were no *a priori* reasons to believe that the results of our study would be biased by seasonal differences in rainfall. Recorders were attached to trees at a height of approximately 2 meters above ground and recorded continuously for the duration of the study, or until battery and/or unit malfunction. We recorded at a sampling rate of 16 kHz, 16-bit resolution and a gain setting of 40 dB, giving a Nyquist frequency of 8 kHz, which is well above the range of Bornean gibbon calls (0.4 to 1.8 kHz). Recordings were saved as consecutive two-hour Waveform Audio File Format (WAV) with a size of approximately 230 MB. Gibbon calls can be heard by human observers up to about 1 kilometer depending on topography^32^, but the recording distance for autonomous recording devices is often substantially less than that. For example, early field trials indicated that the maximum gibbon recording distance on our recorders and in this environment was 350–400 m, but calls detected at >400 meters were of very low-quality (<10 SNR) and difficult to distinguish using aural and visual means, which means that there was a low possibility of recording the same calls on multiple recorders. Home range size for two gibbon groups in our population was previously estimated to be 34 ha (0.34 km^2^), but home range size in gibbons has been shown to be highly variable even within populations^55^, so this may not be representative of the gibbons included in our study. But, given the high degree of territoriality in gibbons^16^, and their relatively small home range size relative to grid spacing of the autonomous recorders, the possibility of re-recording the same vocalizing pair on different recorders in the present study was minimal.

### Acoustic analysis

**Figure 6:**
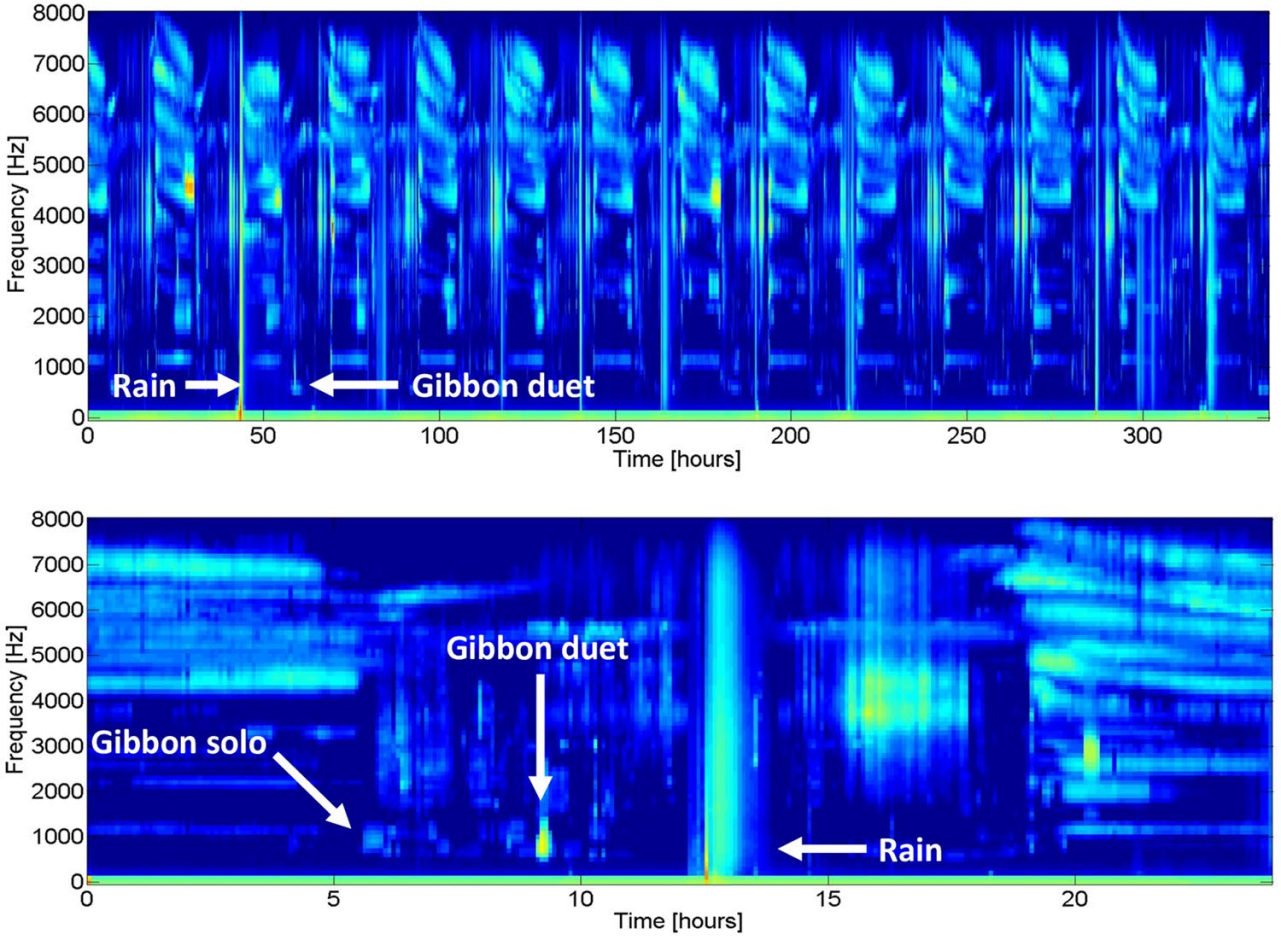
Long-term spectral average (LTSA) plots from a single recording location in Danum Valley Conservation Area over a two-week period (top) and over a 24-hour period starting at 00:00 (bottom). LTSAs are essentially spectrograms that are averaged over a much longer time period than traditional spectrograms. The LTSAs shown here have a temporal and spectral resolution of ∆t = 300 s and ∆f = 100 Hz respectively. Note the relative lack of acoustic activity in the gibbon frequency range.

**Figure 7:**
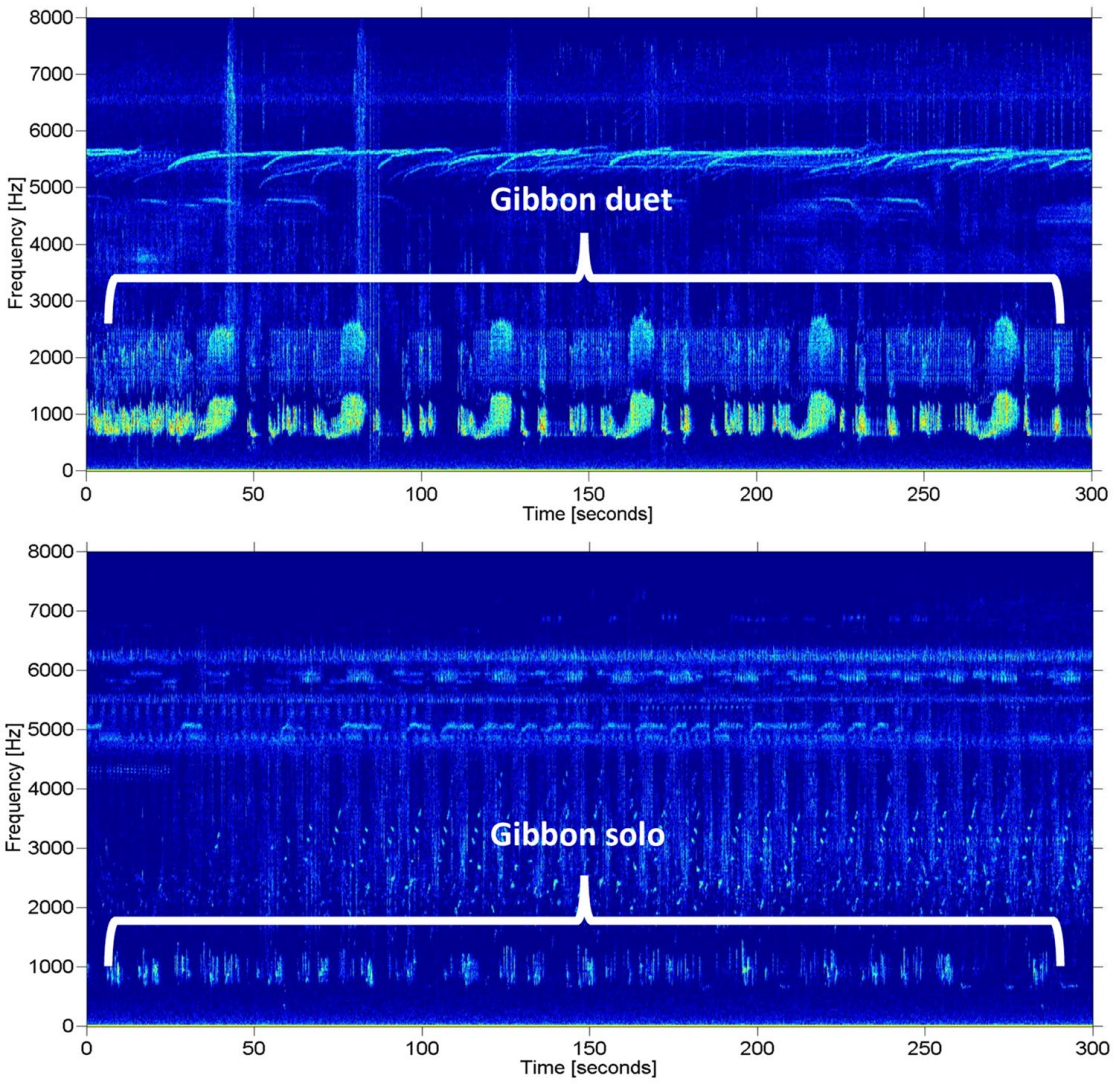
Representative spectrograms of a Bornean gibbon duet (top) and a male solo (bottom). The relative difference in amplitude, and the presence of harmonics in the duet, indicate that the duetting animals were closer to the autonomous recorder than the soloing male. Spectrograms were made with a 1000-point Hann window and 0% overlap. Background noise and harmonics were not removed.

We created long-term spectral averages plots (LTSAs) using the Matlab-based program Triton^63^ at a temporal and spectral resolution of ∆t = 300 s and ∆f = 100 Hz, respectively. A single observer (DJC) identified gibbon calling events using a combination of visual and aural inspection of 24-hour periods, and documented the start and stop time of the calling event, whether the calling event was a male solo or a duet, and the start and stop time of rain events. We were not able to isolate calling bouts during periods of rain, so did not include mornings in which there were substantial, long-lasting rain events in our analysis. A representative LTSA for a two-week period and a 24-hour period is shown in Fig. 6, and a representative spectrogram of a duet between a male and a female, and a male solo is shown in Fig. 7.

### Statistical analysis

Our analyses focused on two separate aspects of gibbon calling behavior, the probability of gibbon calling event on a particular day, and the start time of calls (either solos or duets). For gibbon calling events we included a binary variable (whether there was a gibbon calling event or not) for each recorder on each day. Since we did not know individual or group identity, we could not include number of calls or calling events per day, as it would violate the assumptions of independence for our models^64^, if we were to include two calling events from the same individual or group recorded on the same recorder on the same day.

The climatic variables we used were collected daily at the Danum Valley Field Centre (DVFC) weather station, located in the center of our recording array. The values we included were as follows: maximum temperature in DVFC (°C), minimum temperature in DVFC (°C), humidity in DVFC at 08:00 (%), total rainfall in DVFC (mm per 24-hour period collected every day at 08:00). Weather data were accessed on 10/22/2019 from the South East Asia Rainforest Research Partnership (SEARRP) website^65^. We also extracted information on rain from the LTSAs, including duration of rain events and the time between the last rain event and a gibbon calling event. All continuous outcome and predictor variables were scaled prior to model selection.

To test our hypothesis that the presence of gibbon calling on a particular day is nonrandom with respect to environmental predictors, we created a series of hierarchical models with the presence of gibbon calls (a binary variable indicating presence or absence) on a particular day as the response variable, and a combination of the environmental variables as the predictors. Each model represented a specific hypothesis regarding the relationship between our predictors and outcome variables. As we were modeling a binary outcome, we fit the models using a binomial distribution.

We created a second set of models wherein the start time of the male solo or the duet relative to nautical dawn was the response variable, and a combination of the environmental variables was included as the predictors. We used the R package “suncalc” to obtain the time of nautical dawn for each of our recording days^66^. The “suncalc” package offered a choice between nautical and civil dawn, and males often being calling before sunrise (Clink, personal observation), so we chose nautical dawn as it was the earlier of the two. We used the R package “lunar” to obtain the lunar phase^67^. A summary of the outcome and predictor variables is shown in Table 3.

**Table 3:** Summary of outcome variables, predictor variables and random effects included in models.

Outcome variable	Description	Mean and SD	Range
Gibbon calling event	A binary variable indicating presence of gibbon calls on a particular day.	~	
Solo start time	Minutes relative to nautical dawn until the start of the male solo.	26.6 ± 42.2	-12.8–263.9
Duet start time	Minutes relative to nautical dawn until the start of the duet.	135.0 ± 52.5	28.0–308.4
**Predictor variable**			
Maximum Temperature	Daily maximum temperature (°C)	31.4 ± 1.4	25.4–34.3
Minimum Temperature	Daily minimum temperature (°C)	23.4 ± 0.7	22.2–25.4
Humidity	Daily average of relative humidity (%).	96.2 ± 2.2	85.0–99.0
Rainfall previous day	Total precipitation (mm/24 h) taken at 08:00 each day.	6.9 ± 7.1	0.0–31.7
Time since rain event	The time (h) between the last rain event and gibbon calling (for timing models only)	2.7 ± 4.5	0.0–15.0
Night rain	Binary variable indicating the presence of rain (any amount) from 18:00–06:00 the night preceding gibbon calling.	~	
Lunar cycle	Categorical variable indicating whether the moon was full, new, waxing or waning.	~	
**Random effects**			
Recorder	Recorder on which the calling event occurred.	~	

For all models we used the R package “lme4” to build the hierarchical models, and compared the models using an information criterion approach (Akaike’s Information Criterion adjusted for small sample sizes, AICc^68^). We considered predictors reliable if their 95% confidence intervals did not overlap zero. All models (calling event and calling start time) included a random effect for recorder. To estimate the amount of variance explained by the predictor variables and random effects in our top models we calculated a pseudo-R^2^ value^69^ using the “MuMIn” R package^70^. A complete list of the models used is included in Online Supporting Material.

### Ethical statement

The research presented here adhered to all local and international laws. Institutional approval was provided by Cornell University (IACUC 2017–0098). Sabah Biodiversity Centre and the Danum Valley Management Committee provided permission to conduct the research.

## Supplementary information

Supporting Information.

Supporting Information.

Supporting Information.

Supporting Information.

Supporting Information.

## Data Availability

All data and R code needed to recreate analyses presented in the paper are available as online supporting information. Access to raw sound files will be provided upon reasonable request to the corresponding author.
